# Using a transcriptome sequencing approach to explore candidate resistance genes against stemphylium blight in the wild lentil species *Lens ervoides*

**DOI:** 10.1186/s12870-019-2013-6

**Published:** 2019-09-11

**Authors:** Zhe Cao, Li Li, Karan Kapoor, Sabine Banniza

**Affiliations:** Crop Development Centre / Department of Plant Sciences, University of Saskatchewan, Saskatoon, Saskatchewan S7N 5A8 Canada

**Keywords:** RNA-Seq, *Stemphylium botryosum*, Disease resistance, Histopathology

## Abstract

**Background:**

Stemphylium blight (SB), caused by *Stemphylium botryosum*, is a devastating disease in lentil production. Although it is known that accessions of *Lens ervoides* possess superior SB resistance at much higher frequency than the cultivated lentil species, very little is known about the molecular basis regulating SB resistance in *L. ervoides*. Therefore, a comprehensive molecular study of SB resistance in *L. ervoides* was needed to exploit this wild resource available at genebanks for use by plant breeders in resistance breeding.

**Results:**

Microscopic and qPCR quantification of fungal growth revealed that 48, 96, and 144 h post-inoculation (hpi) were interesting time points for disease development in *L. ervoides* recombinant inbred lines (RILs) LR-66-637 (resistant to SB) and LR-66-577 (susceptible to SB). Results of transcriptome sequencing at 0, 48, 96 and 144 hpi showed that 8810 genes were disease-responsive genes after challenge by *S. botryosum*. Among them, 7526 genes displayed a similar expression trend in both RILs, and some of them were likely involved in non-host resistance. The remaining 1284 genes were differentially expressed genes (DEGs) between RILs. Of those, 712 DEGs upregulated in LR-66-637 were mostly enriched in ‘carbohydrate metabolic process’, ‘cell wall organization or biogenesis’, and ‘polysaccharide metabolic process’. In contrast, there were another 572 DEGs that were upregulated in LR-66-577, and some of them were enriched in ‘oxidation-reduction process’, ‘asparagine metabolic process’ and ‘asparagine biosynthetic process’. After comparing DEGs to genes identified in previously described quantitative trait loci (QTLs) for resistance to SB, nine genes were common and three of them showed differential gene expression between a resistant and a susceptible bulk consisting of five RILs each. Results showed that two genes encoding calcium-transporting ATPase and glutamate receptor3.2 were candidate resistance genes, whereas one gene with unknown function was a candidate susceptibility gene.

**Conclusion:**

This study provides new insights into the mechanisms of resistance and susceptibility in *L. ervoides* RILs responding to *S. botryosum* infection. Furthermore, we identified candidate resistance or susceptibility genes which warrant further gene function analyses, and which could be valuable for resistance breeding, if their role in resistance or susceptibility can be confirmed.

**Electronic supplementary material:**

The online version of this article (10.1186/s12870-019-2013-6) contains supplementary material, which is available to authorized users.

## Background

The cultivated lentil (*Lens culinaris* Medikus ssp. *culinaris*) is one of the most agronomically important grain legumes with a global production of 6.3 million tons in 2016 which was primarily contributed by Canada (50.8%), India (16.8%), Turkey (5.8%) and the USA (4.0%) [1]. Abiotic and biotic stresses are reducing the yield potential of lentil, so breeding efforts have focused on the development of varieties with resistance to these stresses. The main breeding strategy has been relying on selection of superior individuals derived from crosses between elite breeding lines or cultivars. Due to the intensive selection driven by certain traits of interests and hybridization of closely related breeding lines, the genetic variability of lentil germplasm inevitably has been narrow [2]. Associated with limited genetic variability in crops is a heightened risk for crop vulnerability to biotic and abiotic stresses, a major concern shared among plant breeders and growers alike [3]. Stemphylium blight (SB), caused by the necrotrophic ascomycete *Stemphylium botryosum* Wallr., is a devastating disease of lentil in several lentil-producing countries, including Canada, India, the USA, and Australia which together account for over 70% of global production [4, 5]. This pathogen infects plants by airborne conidia that develop successive cycles of conidia on plants. In the early stage of SB, disease symptoms manifest as tan to light brown spots on lentil leaves. As the infection progresses, those initial spots expand to the entire leaves, resulting in the complete drying of leaves followed by defoliation. In the final stages, SB symptoms are also found on stems and hamper nutrient and water transport, which eventually kills the host. The economic losses caused by this pathogen can be up to 80% [6]. A few SB resistant commercial cultivars have been developed in some of the lentil-producing regions, especially in Bangladesh (reviewed in [7]).

Studies in a range of crop species have shown that many alleles representing broad genetic diversity and phenotypic variation reside in wild germplasm [8]. Exploring novel disease resistance alleles from exotic libraries has been a useful strategy for genetic improvement in many crops [3, 9]. In lentil, Podder et al. [7] evaluated resistance to SB among 70 lentil accessions representing seven species in the genus *Lens* and found that *L. ervoides* (Brign.) Grande exhibited superior SB resistance in a much higher frequency than other species. Facilitated by the sophisticated ovule rescue technique that overcomes the interspecific reproductive barriers between *L. ervoides* and *L. culinaris* [10], interest in introgressing useful genes from *L. ervoides* to elite cultivars has been increasing strongly, in particular because resistance to other diseases such as ascochyta blight (caused by *Ascochyta lentis* Vassiljevsky) and anthracnose (caused by *Colletotrichum lentis* Damm) has also been identified in this species [11].

To date, detailed knowledge of the molecular bases underlying SB resistance in *L. ervoides* is still lacking and the only available information is the presence of three quantitative trait loci (QTLs) associated with SB on a single nucleotide polymorphism (SNP) -based linkage map of an F_9_ recombinant inbred line (RIL) population of *L. ervoides* [11]. Despite the identification and localization of these QTLs, their direct use in marker-assisted selection is limited and identification of candidate resistance genes is difficult because those QTL intervals (2-LOD) are relatively large (6 to 21 MB) and contain hundreds of genes [11].

A functional understanding of SB resistance has been largely developed in model plants such as tomato (*Solanum lycopersicum* L.) and *Arabidopsis thaliana* (L.) Heynh. In tomato, Yang et al. [12] mapped a single dominant locus in a 260 Kb chromosomal region conferring resistance to SB (caused by *S. lycopersici* (Enjoji) W. Yamam.) and identified two putative resistance genes coding for a cysteine-rich receptor-like kinase (CRK) and a receptor-like kinase (RLK). This indicated that resistance in tomato was likely mediated via the recognition between plant and pathogen. In *A. thaliana*, Di et al. [13] reported that a gene encoding polygalactoronase-inhibiting protein (PGIP) was upregulated during the *S. solani* infection period. As PGIP belongs to a large family of leucine-rich repeat (LRR) proteins, it was suspected that a hypersensitive response (HR) may affect SB resistance in *A. thaliana* [13]. However, due to the highly complex nature of resistances to necrotrophic pathogens, it is still unknown if these mechanisms can be extended to other plant species. Therefore, a comprehensive molecular study of SB resistance in *L. ervoides* would not only benefit lentil breeding but also broaden our knowledge of SB resistance in systems other than model plant systems.

In recent years, the study of RNA has been significantly improved by the advancement in high-throughput next-generation sequencing techniques that generate massive amounts of data suitable for in-depth quantification of genome-wide gene expression across treatments, time points and genotypes [14]. In lentil, several RNA-sequencing (RNA-Seq) studies were performed to profile transcriptomes, develop molecular markers or investigate plant responses to various biotic and abiotic stresses [15–19]. Most of these studies were conducted by de novo assembly without the aid of a reference genome. The common issues with de novo assembly have been alignment errors, problems in reconstruction of full-length transcripts, and chimerism errors [20]. As the *L. culinaris* genome was recently constructed by Bett et al. [21], reference-based transcriptome assembly is possible and encouraged to improve downstream analyses.

As RNA transcription is a highly dynamic process, the appropriate sampling time is of high importance to capture genes of interest, and to achieve this, the quantitative assessments of fungal development implemented by microscopic observation and quantitative polymerase chain reaction (qPCR) are promising options. Several studies proved that these two methods well integrate both visual and digital assessments of fungal biomass and development in a range of hosts [22–24]. In this study, we recruited those methods and RILs from the F_9_ *L. ervoides* RIL population LR-66 [11] to study SB resistance in *L. ervoides*. Firstly, we performed microscopic and qPCR studies to quantify the development of *S. botryosum* in plants during the first 10 days post-inoculation. Secondly, we conducted a time-series RNA-Seq experiment on two SB infected RILs that displayed contrasting SB susceptibilities. Gene expression data were submitted to differentially expressed gene analyses (DEG) to understand the regulatory pathways that were involved in SB defense responses. To further screen the candidate genes governing resistance and susceptibility to SB, we then performed bulk segregation gene expression analysis using pooled susceptible and resistant RILs from the LR-66 population to verify the expression of those DEGs that were located in identified QTLs [11]. The objectives of this study were to extend our understanding of SB resistance and identify putative resistant genes to facilitate use of *L. ervoides* in commercial lentil breeding.

## Results

### Quantification of fungal development

**Fig. 1:**
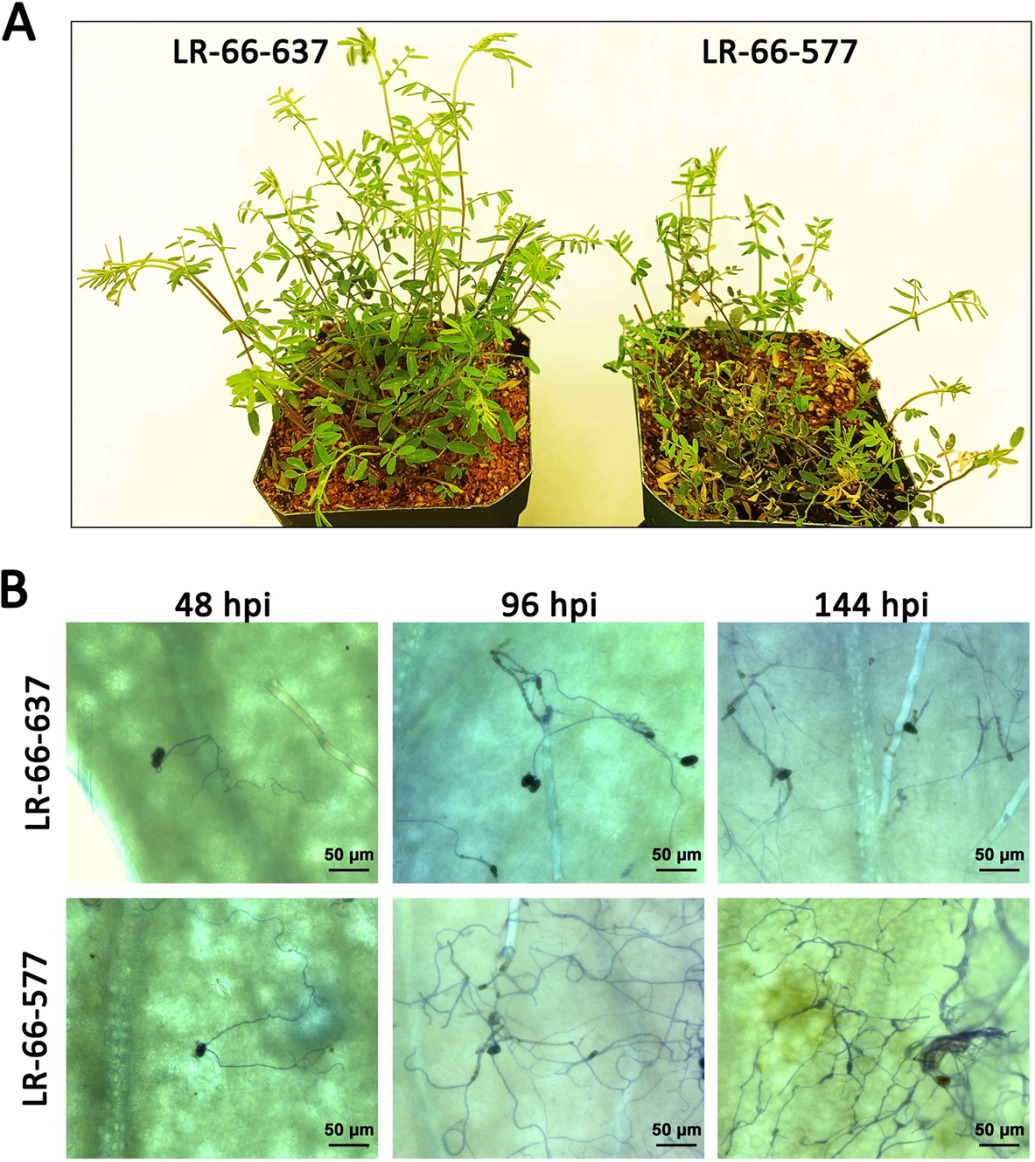
The distinct differences in the level of resistance to *Stemphylium botryosum* (isolate SB19) between the *Lens ervoides* RILs LR-66-637 and LR-66-577. **a** Lateral view of the resistant RIL LR-66-637 (left) and the susceptible RIL LR-66-577 (right) at 144 hpi. **b** Histopathological overview of SB19 progression at 48, 96 and 144 hpi in the two RILs. Photos were taken at 200x magnification with bright field microscopy

**Fig. 2:**
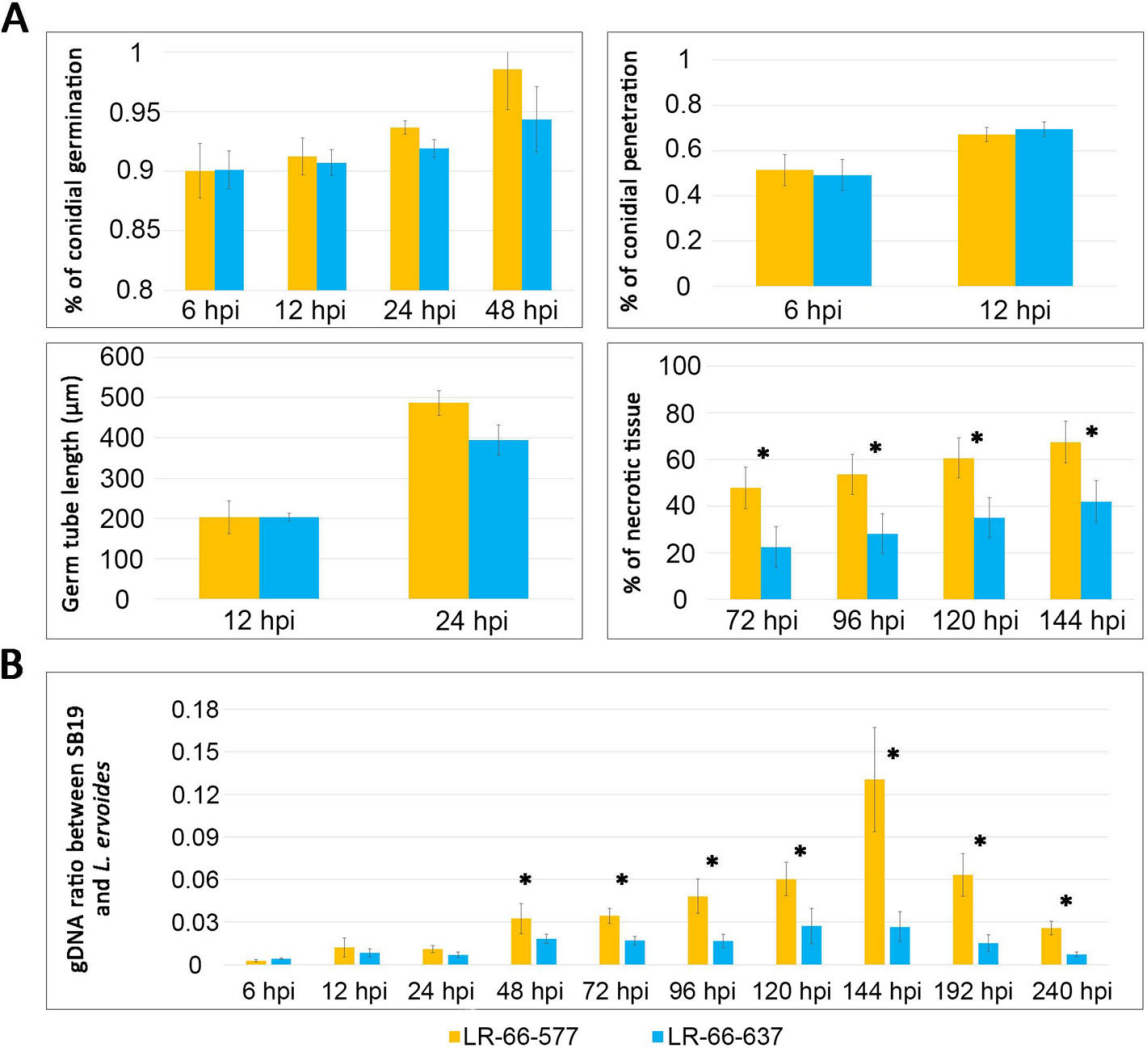
Quantification of *Stemphylium botryosum* (isolate SB19) developmental stages in the resistant *Lens ervoides* RIL LR-66-637 and the susceptible RIL LR-66-577. **a** Assessments through histopathology of SB19 development in two RILs from 24 to 144 hpi. **b** qPCR quantification of relative SB19 biomass in two RILs from 6 to 240 hpi. SB19 biomass was determined by the ratio of SB19 over *L. ervoides* gDNA using qPCR primers of RPL-4 (SB19 specific) and LcEF1a (*L. ervoides* specific). Asterisks indicate significant differences between means at *P* < 0.05. Error bars represent standard errors of the mean

Our inoculation experiment with *S. botryosum* isolate SB19 resulted in distinctly more disease in *L. ervoides* RIL LR-66-577 than LR-66-637 (Fig. 1). The percentage of conidial germination and penetration, and germ tube lengths were recorded for the first 48 h post-inoculation (hpi) (Fig. 2a). Results showed that the germination of conidia increased over time and reached its highest level (94.4% for LR-66-637, 98.5% for LR-66-577) at 48 hpi. Approximate 50% of conidia had germinated with germ tubes that had successfully penetrated into the plant epidermal cells at 6 hpi (both RILs), which increased to 69.6% (LR-66-637) or 67.2% (LR-66-577) at 12 hpi. Germ tube length on the leaf surface was quantified from 12 to 24 hpi and revealed a substantial increase from approximate 200 μm (both RILs) at 12 hpi to 395 μm (LR-66-637) or 486 μm (LR-66-577) at 24 hpi. However, there was no statistical difference between the resistant RIL LR-66-637 and the susceptible RIL LR-66-577 for these fungal growth parameters. At 48 hpi, we started to observe a few chlorotic spots on leaflets. As the infected plants displayed evidence of leaf necrosis at 72 hpi, we decided to record the percentage of necrotic leaf tissue from 72 to 144 hpi at 24-h-intervals. Results showed that necrosis increased with incubation time in both RILs, and the susceptible RIL LR-66-577 had a significantly higher percentage of necrotic leaf tissue than the resistant RIL LR-66-637 throughout this period.

To confirm this trend, we then used qPCR to assess the relative fungal biomass (Fig. 2b). Results showed that fungal growth was slow before 48 hpi but accelerated after 48 hpi. No statistical differences were seen between RILs before 48 hpi, which agrees with our histopathological observations, but significantly more fungal biomass developed in the susceptible LR-66-577 up to the end of observations at 240 hpi. Interestingly, the relative fungal biomass decreased after 144 hpi in both resistant and susceptible RILs, indicating that expansion and growth of lentil plants outpaced fungal growth as a secondary cycle of infection through a new-generation of air-borne conidia of SB19 was largely immobilized due to lack of air currents inside the bags that enclosed single pots to maintain high air humidity. Based on these observations, we decided to collect samples at 48, 96, and 144 hpi for further analysis.

### Analyses of variability among RILs samples at different time points

**Fig. 3:**
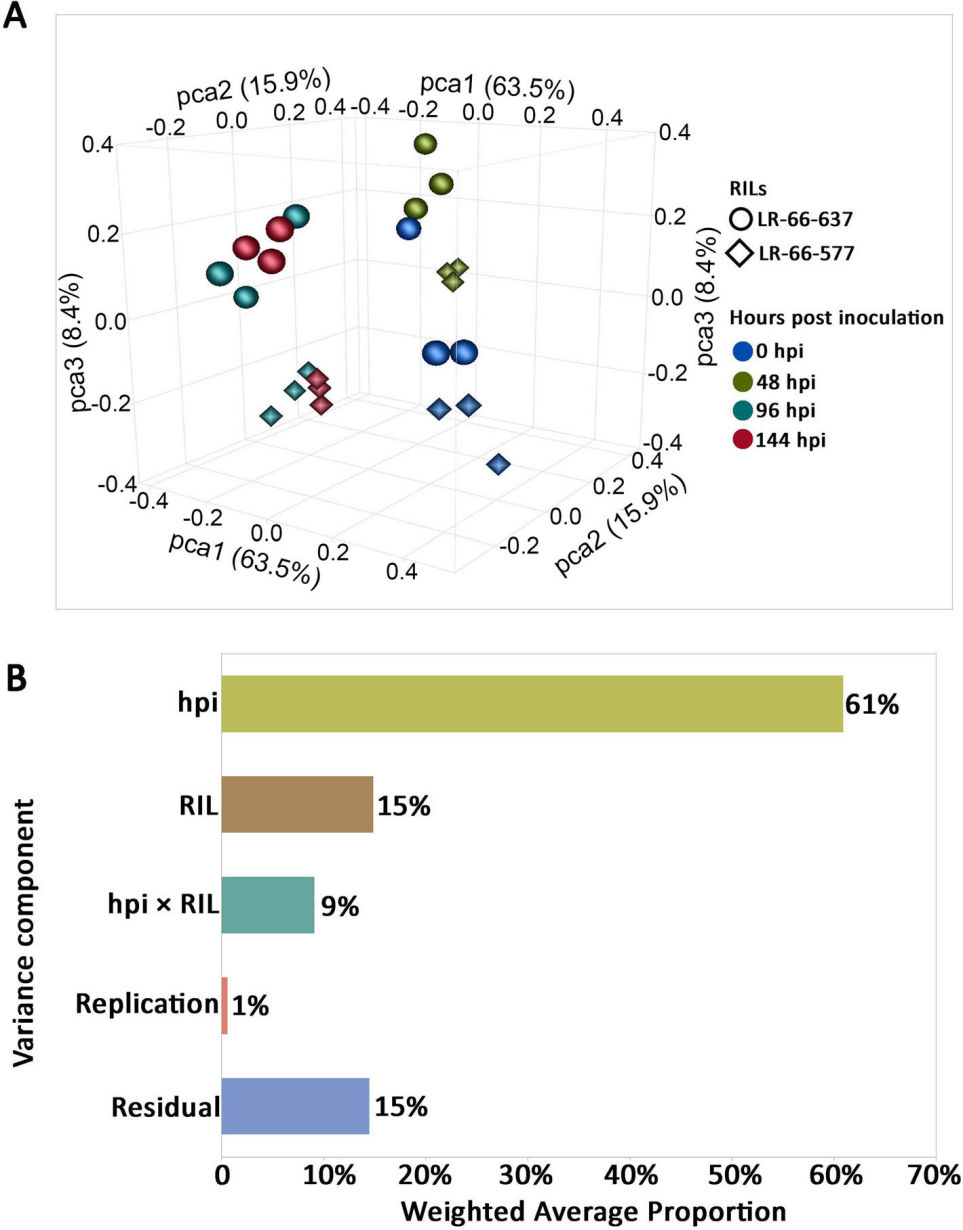
Overview of transcriptome variabilities among 24 *Stemphylium botryosum* (isolate SB19)-infected samples of *Lens ervoides* RILs LR-66-637 and RIL LR-66-577. **a** Principal component analysis of samples. Each point on the PCA plot represents an individual sample. **b** Partition of main effects of hours post inoculation (hpi), RILs, and the interaction of RIL × hpi using principal variation component analysis (PVCA)

To assess the variability of gene expression of RIL samples at different time points, we performed principal component analysis (PCA) to reduce the data dimensionality for ease of visualization. This revealed that the three replicates of samples clustered closely together after excluding one replicate of LR-66-637 at 0 hpi which deviated from other replicates. This sample was considered as an outlier and was therefore discarded from the subsequent analyses. After removing this outlier, the PCA plot clearly separated samples of the same RIL at 0 and 48 hpi, but not at 96 and 144 hpi which were located together (Fig. 3a). PCA also separated LR-66-637 from LR-66-577 samples, especially at 96 and 144 hpi.

To further attribute the proportion of variance to known sources of variation, we conducted a principal variance component analysis (PVCA) on these samples. Results showed that hpi, RILs, and the interaction of RILs × hpi together accounted for 85% of the total variance (Fig. 3b). Among these effects, the largest variance proportion was contributed by hpi (61%), followed by RILs (15%) and the interaction of RILs × hpi (9%). A very small part of the variance (1%) was partitioned to replicates, which was expected. The remaining 14% of variance proportion cannot be explained by the effects of the model and were attributed to the residual.

### Analyses of common disease-responsive genes

The variable transcriptome profiles between LR-66-637 and LR-66-577 at 0 hpi indicated that differentially expressed genes between RILs also correlated with plant characteristics other than disease defense responses. As such, in order to limit the differential gene expression (DGE) analyses to those genes that are likely involved in disease defense responses, we first compared the genes expression at 48, 96, and 144 hpi with that at 0 hpi, which resulted in a total of 8810 genes that displayed differential gene expression (fold change > 2 or FDR < 0.05) during the SB19 infection process between 48 and 144 hpi. The expression heatmap showed that LR-66-637 and LR-66-577 exhibited some similar features in their expression patterns (Additional file 1: Figure S1). These data indicated that a large proportion of disease-responsive genes reprogrammed in a similar manner in the resistant and susceptible RILs after the initial challenge with SB19. To identify these genes, we performed DGE analyses between RILs at 48, 96, and 144 hpi and found that the vast majority of the 8810 disease-responsive genes (7526 genes) were not differentially expressed (fold change < 2 or FDR > 0.05) between RILs. Those genes may be involved in non-host resistance of plants, which is a non-specific and broad-spectrum defense mechanism that is universally present in plants as a defense against microorganisms [25].

**Fig. 4:**
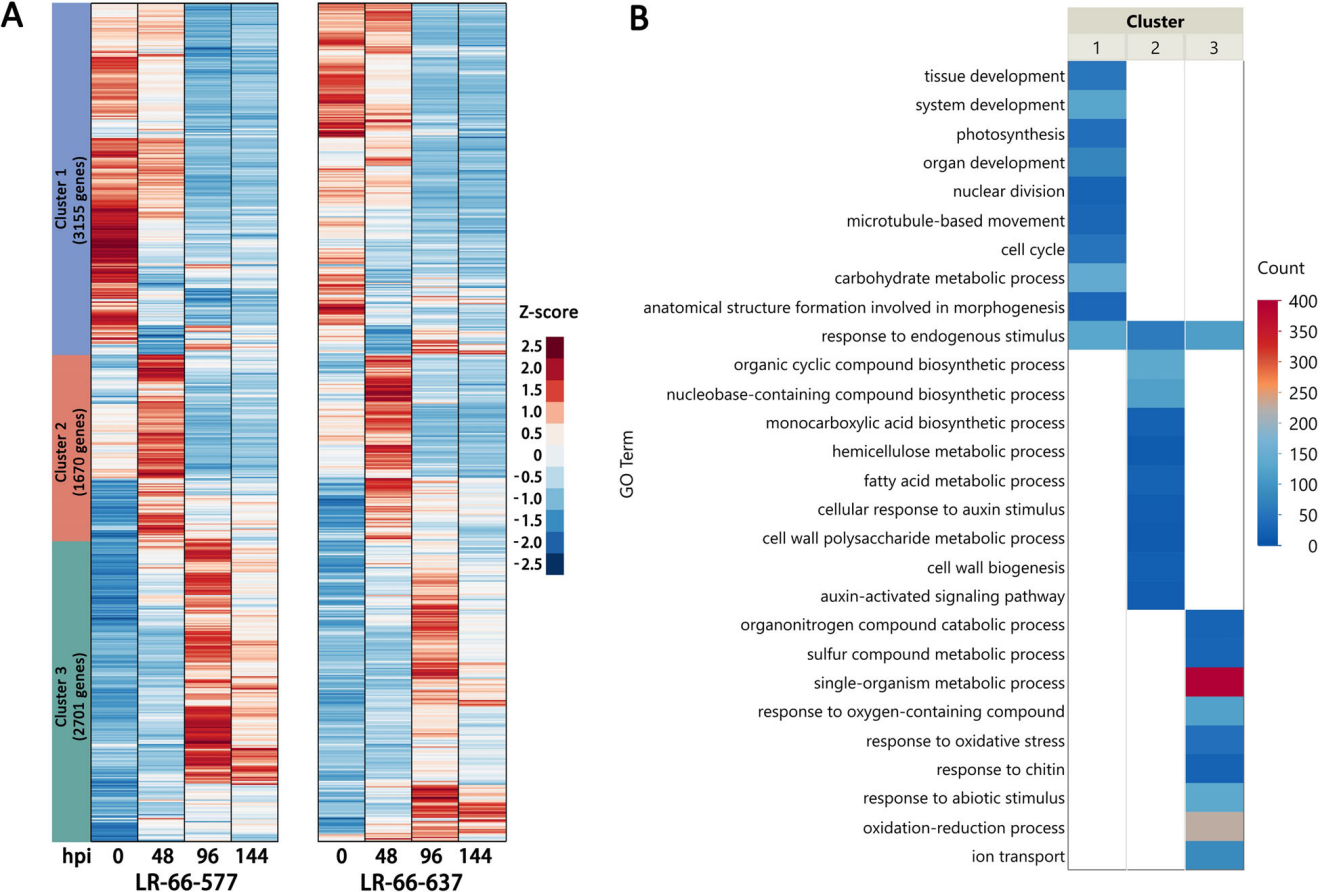
K-mean clustering and GO enrichment analyses of 7526 common disease-responsive genes between the resistant *Lens ervoides* RIL LR-66-637 and the susceptible RIL LR-66-577 after infection with *Stemphylium botryosum* (isolate SB19). **a** Gene expression heatmap of three hierarchically clustered (Ward method) genes. The averaged FPKM of three biological replicates was used to calculate Z-score. **b** Top 10 significant (FDR < 0.05) associated GO terms for three gene clusters

Data of these 7526 genes were submitted to K-mean clustering (Fig. 4a) and Gene Ontology (GO) mapping analyses (Fig. 4b). As a result, three distinct gene clusters were obtained. Cluster 1 consisted of 3155 genes which were primarily downregulated at 96 and 144 hpi. The GO mapping analysis showed that the genes in this cluster were mostly enriched in development-related GO terms such as “tissue development”, “system development”, “organ development”, “nuclear division”, and “anatomical structure formation involved in morphogenesis”, and energy synthesis-related GO terms such as “photosynthesis” and “carbohydrate metabolic process” (Fig. 4b, Additional file 2: Table S1 and Additional file 3: Table S2).

Clusters 2 and 3 contained 1670 and 2701 genes, respectively. Cluster 2 showed a peak of gene expression at 48 hpi, whereas Cluster 3 displayed peaks at 96 and 144 hpi. The most enriched GO terms for Cluster 2 were cell wall-related processes such as ‘hemicellulose metabolic process’, ‘cell wall polysaccharide metabolic process’, ‘fatty acid metabolic process’ and ‘cell wall biogenesis’, indicating that both RILs underwent dramatic cell wall modifications during the early infection period. Disease-responsive genes in Cluster 3 relating to “response to oxidative stress”, “response to oxygen-containing compound”, and ‘oxidation-reduction process’ indicated that reactive oxygen species (ROS) were promoted in both RILs during the late SB19 infection process.

### Analyses of genes differentially expressed between RILs

**Fig. 5:**
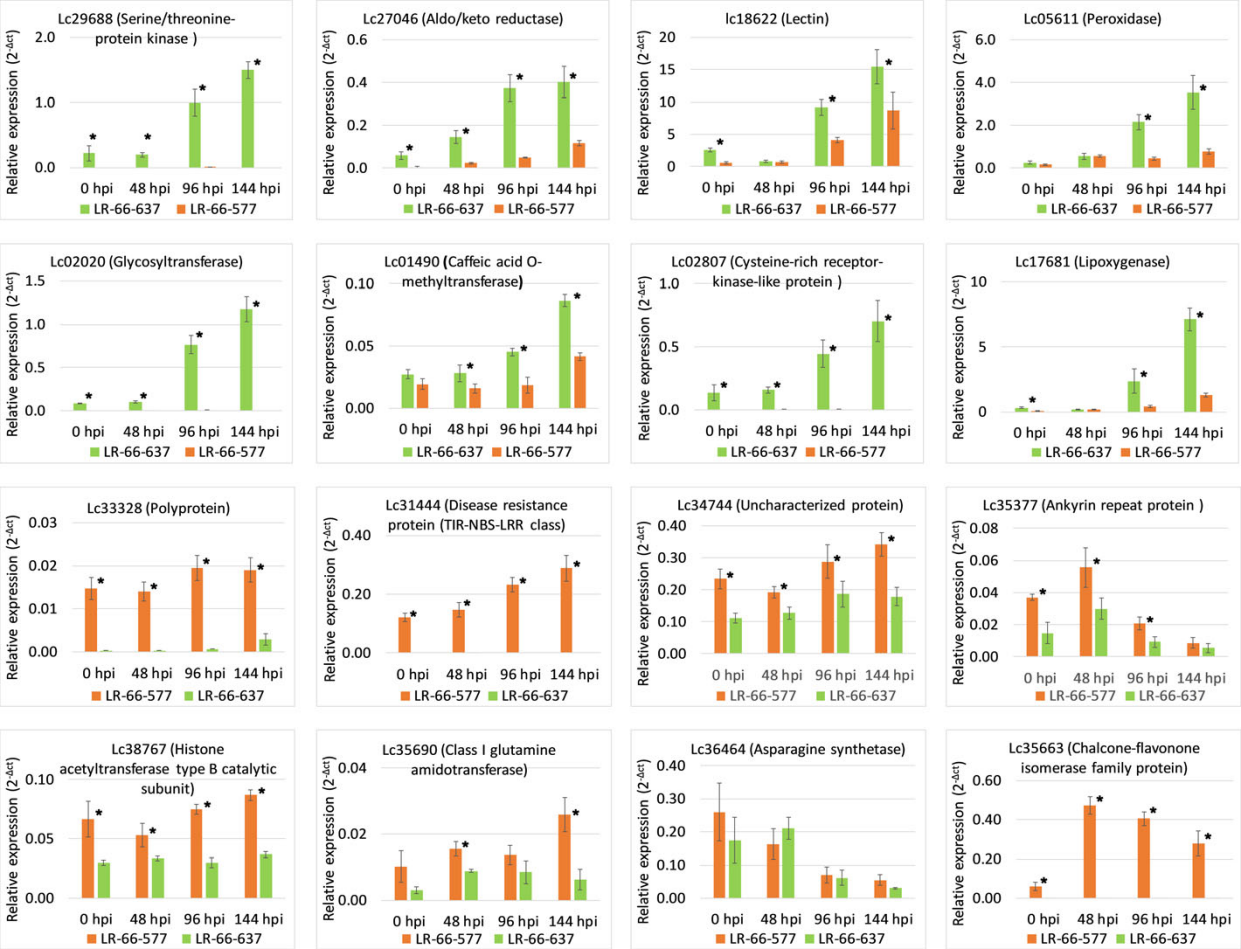
qPCR validation of arbitrarily selected 16 differential expressed genes identified in resistant *Lens ervoides* RIL LR-66-637 and susceptible RIL LR-66-577 after infection with *Stemphylium botryosum* (isolate SB19). Asterisks indicate the significant difference between means at *P* < 0.05. Error bars represent standard error

Among the 8810 disease-responsive genes, 1284 genes were differentially expressed (fold change > 2, FDR < 0.05) between LR-66-637 and LR-66-577 at 48, 96 and 144 hpi (Additional file 4: Table S3). We arbitrarily selected eight of these DEGs upregulated in LR-66-637 and another eight DEGs upregulated in LR-66-577 for qPCR verification in an independent SB19 inoculation experiment (Fig. 5). All but one (94%) exhibited expression trends consistent with those of the RNA-Seq experiment, indicating that expression of DEGs is reproducible across independent experiments.

**Fig. 6:**
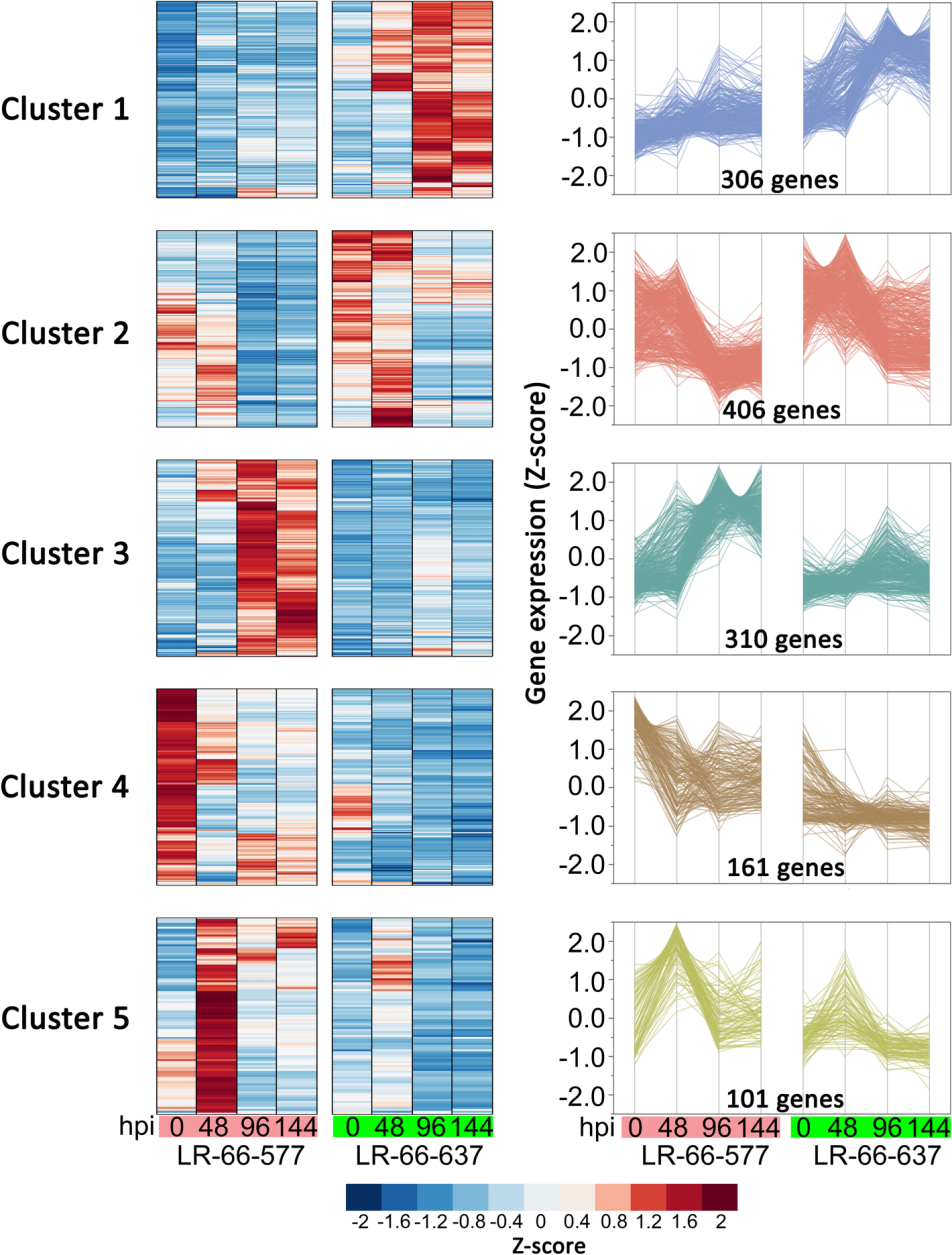
K-mean clustering (Ward method) of 1284 differentially expressed genes between resistant *Lens ervoides* RIL LR-66-637 and susceptible RIL LR-66-577 after infection with *Stemphylium botryosum* (isolate SB19). The identified five major gene clusters are visualized in heatmap (left) and line graph (right)

For a better visualization of genes, the 1284 DEGs were hierarchically clustered into five expression clusters (Fig. 6). Clusters 1 (306 genes) and 2 (406 genes) comprised genes that were upregulated in the resistant RIL LR-66-637, whereas 310 genes in Cluster 3, 161 in Cluster 4 and 101 genes in Cluster 5 were upregulated in the susceptible RIL LR-66-577. Based on their expression patterns, it was hypothesized that the 712 genes in Clusters 1 and 2 are correlated with the enhanced resistance to *S. botryosum* in LR-66-637, and the 572 genes in Clusters 3, 4 and 5 are associated with susceptibility to the pathogen in LR-66-577.

**Fig. 7:**
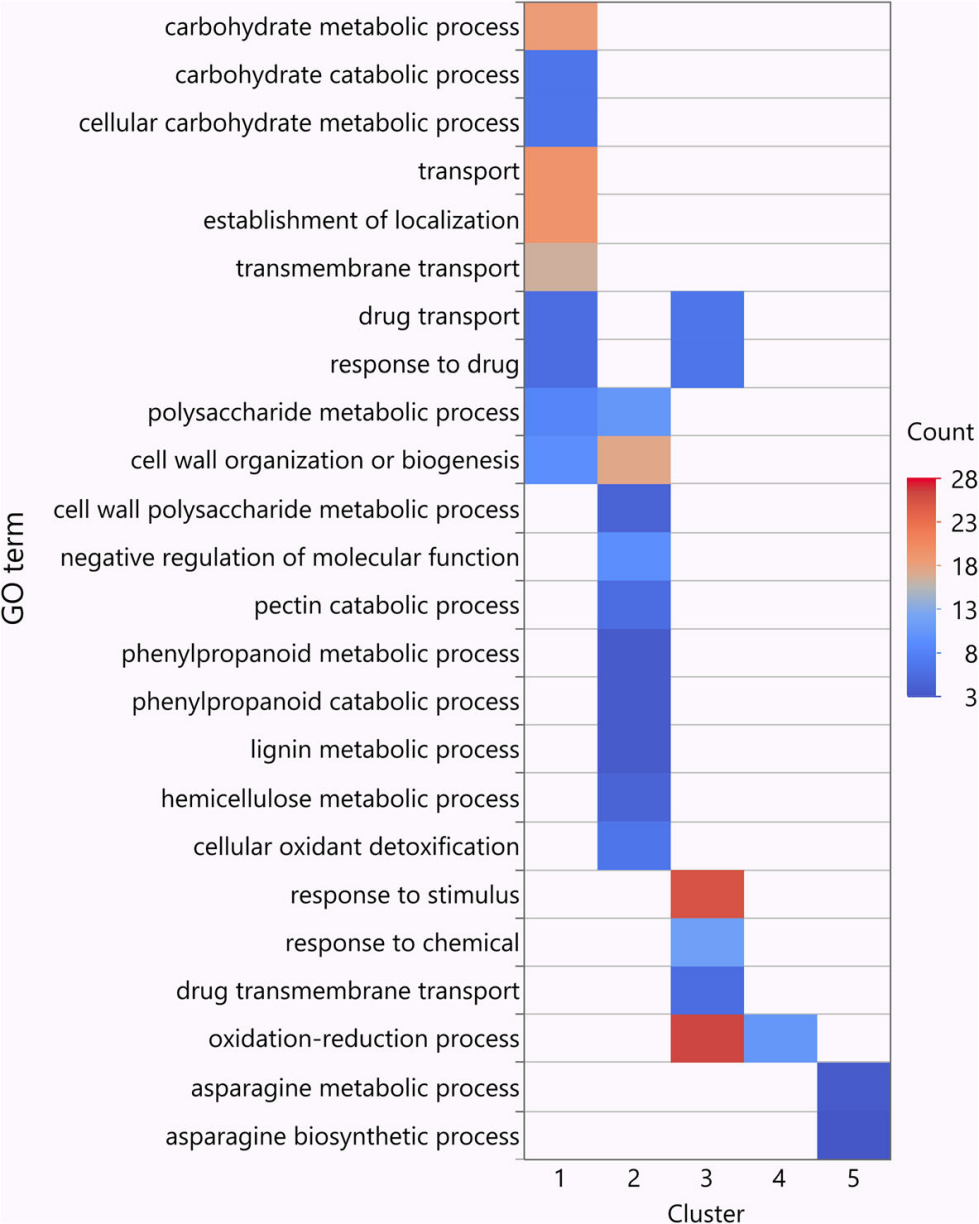
Significant (FDR < 0.1) associated GO terms for five differentially expressed genes clusters identified from samples of resistant *Lens ervoides* RIL LR-66-637 and susceptible RIL LR-66-577 after infection with *Stemphylium botryosum* (isolate SB19)

To understand their biological relevance, we performed GO enrichment analyses for those DEGs in each of the clusters (Fig. 7, Additional file 5: Table S4 and Additional file 6: Table S5). Genes in Cluster 1 were primarily enriched in those involved in a variety of primary metabolic and transportation processes including ‘carbohydrate metabolic process’, ‘cellular carbohydrate metabolic process’, ‘polysaccharide metabolic process’, ‘transport’, ‘establishment of localization’, and ‘transmembrane transport’. In Cluster 2, a large number of genes were enriched in several cell wall related processes such as ‘cell wall organization or biogenesis’, ‘cell wall polysaccharide metabolic process’, ‘pectin catabolic process’, and ‘hemicellulose metabolic process’. In Cluster 3, the majority of genes were enriched for those associated with immune responses such as ‘oxidation-reduction process’, ‘response to stimulus’, and ‘response to chemical’. For the remaining 262 genes in Clusters 4 and 5, HR-associated GO function of ‘oxidation-reduction process’, and two PCD-related GO terms of ‘asparagine metabolic process’ and ‘asparagine biosynthetic process’ were identified, indicating a higher level of HR activity in LR-66-577 during the early SB19 infection [26].

### Screening of candidate resistance genes using QTL and bulk segregant gene expression analyses

**Fig. 8:**
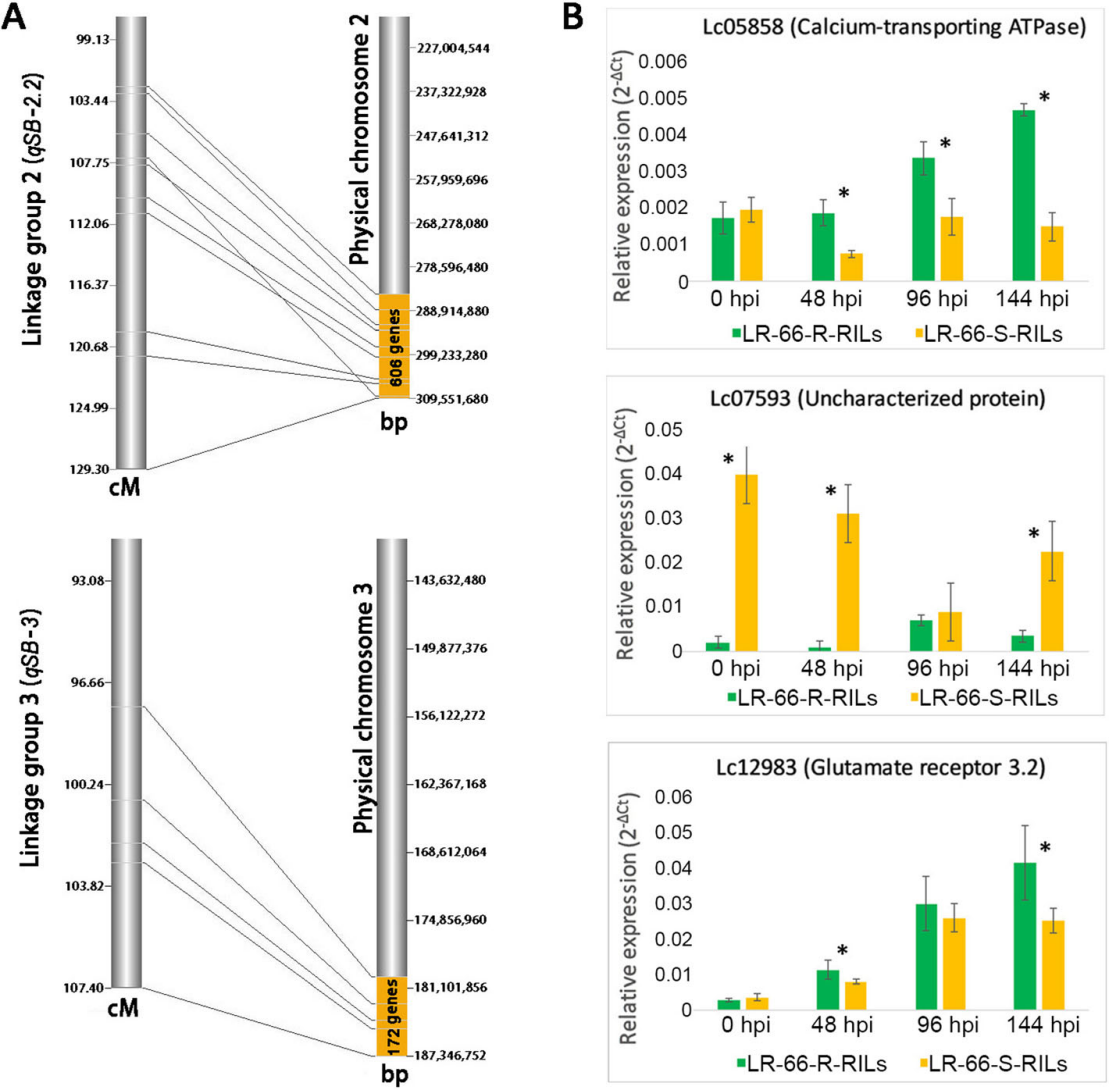
Bulk segregation gene expression analysis of three genes that co-localize within two putative *Stemphylium botryosum* (isolate SB19) resistance QTL intervals on the genetic map of *Lens ervoides*. **a** Projection of QTL brackets from linkage group to the physical chromosomal regions. The linkage maps (left) show the arrangement of SNP markers distributing in 2-LOD QTL intervals (Bhadauria et al., 2017). The right column shows the projected QTL putative regions (yellow) in the corresponding physical chromosomal regions. **b** Expression profiling of three genes between pools of SB-resistant and susceptible RILs. Lc05858 and Lc07593 are in *qSB-2.2* QTL region, and Lc12983 is in *qSB-3* interval. Asterisks indicate the significant difference between means at *P* < 0.05. Error bars represent standard error

Previously, three significant QTL intervals associated with SB19 resistance were identified in a SNP-based linkage map of RIL population LR-66 [11]. To narrow down the resistance or susceptibility gene candidates based on RNA-Seq here, the 1284 DEGs were compared to those in the QTL confidence intervals (2-LOD), which resulted in the identification of nine genes that were up- or downregulated in LR-66-637 at 48, 96 and 144 hpi. Among these nine genes, six genes were localized in the QTL *qSB-2.2* interval and three in the QTL *qSB-3* interval. Expression analysis of those genes in pools of the five most SB-resistant and the five most susceptible RILs revealed that two genes (Lc05858: calcium-transporting ATPase; Lc07593: uncharacterized protein) present in *qSB-2.2* and one gene (Lc12983: glutamate receptor3.2) in *qSB-3* were expressed in a manner similar to that in resistant RIL LR-66-637 and susceptible LR-66-577 based on RNA-Seq analysis (Fig. 8 and Additional file 7: Table S6). Quantitative PCR for another two of these genes (Lc09908 and Lc07065) was inconclusive, and four genes (Lc06015, Lc10098, Lc11261, and Lc13725) displayed similar expression between the two pools, indicating that they are more likely associated with other differences specific to LR-66-637 and LR-66-577.

For Lc07593, it was observed that this gene was highly expressed in the SB19-susceptible RILs compared to the resistant RILs, suggesting that Lc07593 could be a susceptibility gene, upregulation of which seemed to increase SB susceptibility. On the other hand, Lc05858 and Lc12983 could be disease resistance genes, as they displayed a reverse expression trend to that of Lc07593.

## Discussion

A thorough understanding of how pathogens interact with their hosts is essential to identify appropriate times during the infection process for the profiling of the transcriptome. In the present study, the difference in SB19 development between the resistant RIL LR-66-637 and the susceptible RIL LR-66-577 was negligible before 24 hpi, indicating that the resistance toward SB19 in RIL LR-66-637 was probably based on post-penetration inhibition of the fungus. Similar observations were also reported for *S. botryosum* infecting alfalfa where Cowling and Gilchrist [27] found that the development of *S. botryosum* did not differ among the resistant, moderately resistant and susceptible alfalfa clones during the early period of infection. During the period from 48 to 144 hpi, fungal biomass started to accumulate in RILs, suggesting that *S. botryosum* initiated the release of phytotoxins to disrupt host cells around 48 hpi. Biomass accumulation was significantly higher in LR-66-577 than in LR-66-637, possibly as a result of enhanced tolerance to phytotoxins in LR-66-637 compared to LR-66-577 [28]. As such, 48, 96 and 144 hpi were considered to be interesting time points warranting further exploration of gene expression.

PCA and PVCA are useful tools to visualize the overall gene expression variability and to detect outliers among samples [29]. After excluding an outlier in the PCA plot here, separation of those samples collected at 48 hpi from others gathered at 96 and 144 hpi was evident, which clearly reflected that gene reprogramming at 96 and 144 hpi was similar, but was different from 48 hpi. PCA also separated LR-66-637 from LR-66-577 samples, indicating that the transcriptome responses of LR-66-637 and LR-66-577 were different after challenge with SB19. As this separation was more evident at 96 or 144 hpi than at 48 hpi, a significant interaction effect of hpi × RIL was expected. These results confirmed that the transcriptomes of RILs responded to SB19 infection in a time-dependent manner as has been observed in other transcriptome studies [30–32].

Non-host resistance is a non-specific and broad-spectrum defense mechanism that is universally present in plants as a defense against microorganisms [25, 33]. By analyzing those disease-responsive genes of both RILs, we observed that a large number of genes downregulated in both RILs were enriched in a series of development-related and energy synthesis-related GO terms. These results seemed to indicate that SB19 infection disrupted the normal growth and energy synthesis of both RILs regardless of their varying levels of resistance to the pathogen. As the resistance towards necrotrophs in plants is always in an incomplete form, the withdrawal of nutrients by *S. botryosum* from plants normally occurs in all individuals of the host species [34]. Pathogen infection leads to a decrease in photosynthesis at the infection sites, which negatively impacts the energy assimilation and interferes with the normal development and growth of the plant [35]. This has been extensively reported in a variety of diseased plants [36–38]. On the other hand, there were also genes that were upregulated after inoculation, either at 48 hpi or at 96 and 144 hpi. As these genes were mostly enriched in cell wall-related and oxidation-reduction processes, it seemed that cell wall modifications and ROS were promoted at certain time points during the SB19 infection process. Any type of interaction between pathogen and host will trigger some degrees of cell wall modifications [25, 33, 34]. When plants are stressed by pathogens, variable amounts of ROS are released to activate the downstream signal transduction pathways and expression of a series of defense genes [39–41]. The activation of ROS is a common form of non-host resistance that universally occurs in plants, regardless of their difference in the level of disease resistance [42–44].

In addition to these common disease-responsive genes, another 1284 disease-responsive genes were differentially expressed between LR-66-637 and LR-66-577. Some of these genes may contribute to the difference in the level of disease resistance between the two RILs. According to the expression patterns and enriched GO terms of these genes, we found that LR-66-637 mainly upregulated a variety of carbohydrate metabolic, transportation and cell wall related processes. Other gene expression data on the lentil - *S. botryosum* system have not been reported to date, but stronger upregulation of a series of metabolic processes (organic substance metabolic, single-organism metabolic, or cellular glucan metabolic) based on RNA-Seq experiments were reported in the resistant lentil genotypes CDC-Robin, 964a-46, or ILL7537 after inoculation of *Ascochyta lentis,* another lentil pathogen presumed to be a necrotroph [15, 16]. In addition, Yang et al. [45] studied the tomato – *S. lycopersici* system and found that several GO terms of ‘localization’, ‘transporter activity’ and ‘molecular transducer activity’ were activated in the resistant tomato genotype, proposing that the upregulation of these activities were related to *S. lycopersici* resistance. Carbohydrates and transporters are considered essential parts of plant immune systems, with the former generating energy to sustain the continuous production of a series of defense-related metabolites and carbon-based polymers, and the latter fulfilling signal transduction, energy translocation and also extrusion of toxic compounds [46–48]. Theoretically, the promotion of these processes is in accordance with elevated disease resistance against necrotrophs [35, 48, 49]. Morkunas et al. [50] found that a high level of sucrose accounted for the accumulation of a variety of flavonoids which resulted in enhanced resistance in yellow lupin to *Fusarium oxysporum*. Likewise, it was reported that the upregulation of the two plant ATP binding cassette transporters *NpPDR1* and *PEN3,* could enhance disease resistance to the necrotrophic pathogens *Botrytis cinerea* and *Plectosphaerella cucumerina* in *Arabidopsis*, respectively [49, 51].

The promotion of several cell wall related processes suggested that LR-66-637 initiated additional cell wall modification compared to LR-66-577. In the lentil – *A. lentis* system, Khorramdelazad et al. [15] also reported the significant enrichment for the GO term ‘cell wall’ in the resistant genotype ILL7537. During attack by necrotrophic pathogens, the cell wall of a host is damaged by a series of cell wall-degrading enzymes released by the pathogen. If such cell wall degradation can be arrested by the host, a resistant phenotype would be expected [52]. In tomato, Miedes and Lorences [53] found that the appropriate level of xyloglucan (a form of hemicellulose) was essential for maintaining cell wall integrity when under attack from *Penicillium expansum*. In *Arabidopsis*, Lionetti et al. [54] found that *B. cinerea* resistance was enhanced through an increase of pectin methyl esterification which disrupted the ability of microbes to degrade the plant cell wall. As such, we hypothesize that the upregulation of genes in cell wall processes, to a certain degree, may compensate for the cell wall damage through infection and thus improve SB resistance in LR-66-637.

In contrast, LR-66-577 appeared to respond to SB19 invasion in a completely different form considering that the majority of genes upregulated in this RIL were enriched for those involved in ‘oxidation-reduction process’. This suggests that redox homeostasis was substantially altered in LR-66-577 after SB19 invasion, which was reported for other systems previously [55]. A change in redox status in the challenged cells could release a variety of reactive oxygen species including singlet oxygen, superoxide radicals and hydrogen peroxide, accumulation of which could trigger the plant’s hypersensitive response and programmed cell death (PCD) to cause leaf necrosis and, eventually, leaf dehiscence [56, 57]. As necrotrophs can thrive in dead tissues, several studies have reported that necrotrophs are able to hijack the plant immune system and use the host HR machinery to boost their virulence [39, 58, 59]. From these results, we hypothesize that HR and PCD were significantly more promoted in LR-66-577 than LR-66-637 during the SB19 infection process, resulting in susceptibility in LR-66-577.

Previous evidences has shown that HR and PCD are largely induced through the activation of R genes [56–59]. In the present study, we observed that a number of TIR-NBS-LRR and CC-NBS-LRR genes were stronger upregulated in LR-66-577 than LR-66-637 during the infection process (Additional file 5: Table S4). In the lentil – *A. lentis* system, Sari et al. [16] found a TIR-NBS-LRR gene that was also significantly upregulated in two susceptible lentil genotypes after inoculation of *A. lentis*. Such results corroborate an association of R genes, cell death and susceptibility after infection by necrotrophic pathogen [39]. However, there was another set of TIR-NBS-LRR, NBS-LRR, and NB-ARC genes that had higher expression in LR-66-637 than LR-66-577, as would be expected for resistant genotypes. Involvement of such R genes was also reported in the lentil *– A. lentis* system, and Khorramdelazad et al. [15] speculated that a stronger upregulation of a NB-ARC gene conferred enhanced resistance in ILL 7537. Taken together, this supports the emerging picture of R-genes as true resistance genes as well as their possible role as susceptibility factors [34].

Comparing DEGs with previously identified resistance QTLs of LR-66 [11], we identified nine resistance gene candidates and explored them further through bulk segregation analysis. In a bulk segregation analysis (BSA), the phenotypic extremes of two pools tend to either differ in the presence of the gene(s) characterizing the phenotype, or differ in the expression level of the gene(s) responsible for the trait [60]. BSA coupled with gene expression studies has been used frequently to identify candidate genes in varieties of plant species (e.g. [61–63]). BSA was used here and revealed a calcium-transporting ATPase (Lc05858) and a receptor3.2 (Lc12983) with a high expression in the resistant pool indicating that they could be putative resistance genes. A calcium transporting ATPase, such as Lc05858, could play an important role in calcium influx and efflux regulation to maintain an appropriate cellular calcium homeostasis, the change of which is thought to initiate calcium signaling that triggers a series of downstream responses influencing plant growth, development and responses to biotic and abiotic stresses [64–66]. Zhu et al. [67] reported that the expression of a gene coding calcium ATPase decreased HR in *Pseudomonas syringae-*infected tomatoes as *NbCA1* (ER-localized type IIB calcium ATPase)-silenced plants exhibited accelerated programmed cell death (PCD) compared to the wild type. Similarly, Boursiac et al. [68] found that the knockout of *ACA4* and *ACA11* (two calcium-ATPases) induced a higher frequency of HR-like lesions by deactivating the calcium signaling in *Arabidopsis* inoculated with *P. syringae*. Based on this it can be speculated that upregulation of Lc05858 during SB19 infection results in the activation of calcium transportation and the suppression of HR and PCD. Upregulation of the glutamate receptor3.2 Lc12983 in the resistant RILs compared to the susceptible RILs at 48 and 144 hpi may also affect calcium regulation, highlighting its potential importance in the lentil –*S. botryosum* interaction. A previous study showed that a glutamate receptor is mainly responsible for glutamate-dependent membrane depolarization and calcium transportation [69], and Kang et al. [70] and Manzoor et al. [71] observed that the upregulation of glutamate-like receptors AtGLR3.3 or AtGluR3.2 elevated calcium influx and conferred enhanced resistance in *Arabidopsis* against the necrotroph *B. cinerea*. However, further functional studies are required to confirm the role of these genes in resistance.

One gene encoding an uncharacterized protein (Lc07593) was highly expressed in the SB19-susceptible RILs compared to the resistant RILs, suggesting that Lc07593 could be a susceptibility gene. However, this gene is not homologous to any well characterized genes in the database and thus its function is not understood. Future work involving protein purification, structural modeling, and protein-protein interaction analyses may be needed to understand its biological function.

## Methods

### Plant material and inoculum preparation

RILs LR-66-577 (susceptible to SB) and LR-66-637 (resistant to SB) used in this study were transgressive segregants chosen from the *L. ervoides* RIL population LR-66 derived from the cross of L01-827A × IG 72815 developed at the Crop Development Centre at the University of Saskatchewan (Saskatoon, SK, Canada) [11]. Evident transgressive segregation and disease severity in RILs ranging from 11 to 80% in this population indicated that there was fluent genetic variability in SB resistance [11]. Prior to planting, seeds of RILs were scarified to facilitate germination. The scarified seeds were sowed in 10-cm plastic pots filled with Fafard® Germination Mix (Sungro Horticulture® Ltd., Vancouver, BC, Canada). All plants were grown in a growth chamber with a constant temperature of 23 °C and a day length of 16 h light/8 h dark. Only four plants per pot were kept for the subsequent inoculation experiment. The inoculum was prepared from the aggressive *S. botryosum* isolate SB19 which originated from the southeast of Saskatchewan, Canada. The cryopreserved SB19 spores were revitalized on oatmeal V8 agar medium [150 mL V8 juice (Campbell Co., Canada), 10 g Difco™ Potato Dextrose Agar (Becton Dickinson and Co., Franklin Lake, NJ, USA), 3 g calcium carbonate, 850 mL sterile water]. After seven days of incubation at room temperature, conidia were collected from Petri dishes by flooding and scraping the surface of the plates. The conidial suspension was filtered through two layers of miracloth and adjusted to 1 × 10^5^ condia mL^− 1^ using a hemocytometer. Two droplets of Tween 20 (Sigma, Saint Louis, MO, USA) were added to every 1000 mL of suspension before inoculation.

### Pathogen inoculation and experimental design

Four-week old plants were sprayed with the conidial suspension until run off, which was equivalent to approximate 2 to 2.5 mL per plant. Four plants within a pot represented one of three biological replicates and were pooled to generate a biological replicate. Pots were arranged in a completely randomized design in a misting chamber at 100% humidity for the first 48 h to favor germination of conidia. After 48 hpi, those inoculated plants were removed from the misting chamber, each pot was enclosed in a translucent plastic bag, and pots were maintained at 23 °C and 16 h photoperiod. Leaves were sampled at 0, 6, 12, 24, 48, 72, 96, 120, 144, 196, and 240 hpi, immediately frozen in liquid nitrogen and then stored in a -80 °C freezer for future uses.

### Determination of fungal development

Microscopy-based methods and qPCR were used to quantify growth of SB19 in plants. For qPCR, the genes targeting RNA polymerase II second largest subunit (RPL-4) to amplify gDNA of SB19 and elongation factor (LcEF1α) to quantify *L. ervoides* were used (Additional file 8: Table S7). The SDS method [72] was used to extract gDNA from both organisms and a spectrophotometer (NanoDrop™ 8000, Thermo Scientific, Waltham, USA) was employed to quantify the DNA concentration, which was adjusted to 25 ng μl^− 1^ for all samples. Each qPCR reaction contained 2 μl DNA template, 5 μl SYBR® Green (catalog no. 4309155, Thermo Scientific), 0.2 μl each of 10 μM forward and reverse primers, and 2.6 μl molecular grade water. A default fast-run program was used to perform qPCR amplification in a QuantStudio™ 3 System (Applied Biosystems Inc., Foster City, CA, USA). According to the criteria proposed by Weβling and Panstruga [24], the relative fungal biomass was estimated as the proportion of gDNA amplified between RPL-4 and LcEF1α.

For microscopic quantification, six leaflets per biological replicate were collected at each of 6, 12, 24, 48, 72, 96, 120, 144, 196, and 240 hpi. The collected leaflets were cleared in CMAA fixation solution (60% methanol, 30% chloroform, 10% acetic acid) for at least 24 h at room temperature to eliminate chlorophyll in cells. Once treated, they were immersed in a series of decreasing ethanol concentrations of 70% for 1 h, 50% for 1.5 h and 30% for 1.5 h. Staining was performed by immersing leaflets in 0.05% trypan blue overnight. Well stained leaflets were mounted on glass slides in a droplet of 50% glycerol and evaluated under a Zeiss Axioskop 40 microscope (Carl Zeiss, Göttingen, Germany). High-quality photos were generated using a Pixelink A686C camera (Pixelink, Rochester, NY, USA) and Zeiss Axiovision software (Carl Zeiss, Oberkochen, Germany). To quantify fungal development during the incubation process, we record percentage of conidial germination, percentage of conidia resulting in successful penetration (% conidial penetration), and germ tube length. Furthermore, the percentage area of dead tissue per leaflet was visually estimated (% leaf necrosis). Data were analyzed using SAS 9.3 (SAS Institute, Inc., Cary, NC, USA). Normality of errors was assessed with the Shapiro-Wilk test and homogeneity of variance with the Levene’s tests. The mean separation between RILs were conducted using Student’s t-test method (*P* < 0.05).

### RNA sequencing and raw data processing

Samples were collected at 0, 48, 96, and 144 hpi with three biological replicates for LR-66-570 and LR-66-629, for a total of 24 samples. Total RNA extraction was performed from the frozen leaves using the RNeasy Plant Mini Kit (Cat no. 74904, Qiagen Company, Hilden, Germany) following the manufacturer’s instructions. The quality and concentration of RNA was assessed in an Agilent 2100 Bioanalyzer (Agilent Technologies, Santa Clara, CA, USA). Only high-quality RNA samples (RNA integrity number > 7) were used for library construction using TruSeq Stranded Total RNA (Illumina, Inc., San Diego, CA, USA). The constructed libraries were then loaded into a HiSeq 2500 system using TruSeq SBS KIT-HS V4 (Illumina) for 125 bp pair-ended sequencing.

The quality control of returned raw reads was implemented in Trimmomatic (version 0.36) [73] to remove low-quality reads and adaptors under the following parameters: TruSeq3-PE-2.fa:2:30:10, leading:3, trailing:3, slidingwindow:4:15 and minlen:36. The cleaned reads were aligned against the reference genome of *Lens culinaris* V1.2 [21] using STAR (default settings, version 2.6.1a) [74]. As a result, approximate 90% of reads were uniquely aligned to the reference genome for all samples, confirming the high quality of sequencing and mapping (Additional file 9: Figure S2). Gene counting was performed using STAR quantmode during the mapping process. The resulting gene expression data for each sample were first normalized using the fragments per kilobase of transcript per million (FPKM) method before proceeding to principal component analysis (PCA) and principal variance component analysis (PVCA) in the statistical software JMP Genomics 8.0 (JMP Genomics®, SAS Institute).

### Identification of differentially expressed genes (DEGs)

Before comparing the gene expression between RILs, we first conducted comparisons of gene expression in samples from 0 hpi with those collected at 48, 96, and 144 hpi for each RIL to identify those genes that significantly responded to SB19 infection. These comparisons were performed in the R package DESeq2 [75] using the thresholds of false discovery rate (FDR) < 0.05 and gene expression fold change > 2. Among the identified disease-responsive genes, we then conducted pair-wise comparisons between LR-66-637 and LR-66-577 samples from each time point (48, 96 and 144 hpi). The same thresholds used previously were applied here to declare significant DEGs. The resulting DEGs were submitted to JMP Genomics 8.0 (JMP Genomics®, SAS Institute) for K-means clustering analysis. The genes of each cluster with known *Medicago truncatula* orthologs were then mapped to the Gene Ontology (GO) database using the PANTHER (version 14.1) (http://www.pantherdb.org).

### qPCR validation

To verify the repeatability of gene expression captured in the RNA-Seq experiment, an independently inoculated experiment with LR-66-570 and LR-66-629 was conducted following the same experimental conditions and setup as described before. Infected leaf samples were collected and processed as described before, and 16 DEGs identified from RNA-Seq data analysis and one reference gene (LcEF1α) were used for qPCR amplification (Additional file 10: Table S8). Each PCR reaction consisted of 2 μl DNA template, 5 μl SYBR Green, 0.2 μl of each 10 μM forward and reverse primers, and 2.6 μl molecular grade water. qPCR was performed in a QuantStudio™ 3 System (Applied Biosystems Inc.) using the fast-run program with default settings. The relative expression of each gene of interest was calculated as ${2}^{-\left({CT}_{gene\ of\ interest}-{CT}_{reference}\right)}$ recommended by Livak and Schmittgen [76]. Mean separation between RILs was conducted using the Student’s t-test method (*P* < 0.05).

### Bulk segregant gene expression analysis

Based on the results of the phenotypic evaluation of the LR-66 population [11], the five top SB-resistant RILs (LR-66-526, LR-66-543, LR-66-643, LR-66-658, and LR-66-712) and the five most SB-susceptible RILs (LR-66-594, LR-66-605, LR-66-697, LR-66-706, and LR-66-727) were selected to construct resistant and susceptible bulks, respectively. An inoculation experiment was performed on these RILs following the same experimental and environmental settings, and leaflets of RILs were collected at 0, 48, 96 and 144 hpi as described before. Total RNA extraction and quantification for each RIL, as well as qPCR were conducted using the same method described above. Each individual RIL was treated as one biological replicate in each of the two pools, thus each pool consisted of five biological replicates. qPCR reaction and gene expression estimation were conducted for Lc09908, Lc07593, Lc05858, Lc06015, Lc10098, Lc7065, Lc12983, Lc11261, and Lc13725 which were differentially expressed genes that co-localized with previously described quantitative trait loci (QTLs). The mean separation between pools was conducted using the Student’s t-test method (*P* < 0.05).

## Additional files

Additional file 1 **Figure S1.** Expression heatmap of 8810 disease-responsive genes for resistant *Lens ervoides* RIL LR-66-637 and susceptible RIL LR-66-577 at 0, 48, 96 and 144 hpi with *Stemphylium botryosum* (isolate SB19). (JPG 2425 kb)

Additional file 2: **Table S1.** GO enrichment analysis of common stemphylium blight-responsive genes. (XLSX 216 kb)

Additional file 3: **Table S2.** Top enriched GO terms of common stemphylium blight-responsive genes. (XLSX 9 kb)

Additional file 4: **Table S3.** Information of 1284 DEGs identified between *Lens ervoides* RILs LR-66-637 and LR-66-577 across 48, 96 and 144 hpi with *Stemphylium botryosum*. (XLSX 157 kb)

Additional file 5: **Table S4.** GO enrichment analysis of DEGs between *Lens ervoides* RILs. (XLSX 39 kb)

Additional file 6: **Table S5.** Top enriched GO terms of DEGs between *Lens ervoides* RILs. (XLSX 9 kb)

Additional file 7: **Table S6.** DEGs identified by RNA-Seq that co-localized with two stemphylium blight resistance QTL intervals on the genetic map of *Lens ervoides*. Genes were further explored through bulk segeragtion analysis. (XLSX 10 kb)

Additional file 8: **Table S7.** Sequences and amplification efficiencies for *Stemphylium botryosum* and *Lens ervoides-*specific primers. (XLSX 9 kb)

Additional file 9: **Figure S2.** Summary of mapping of 24 libraries on *Lens culinaris* reference genome. (JPG 1023 kb)

Additional file 10: **Table S8.** Primer information for 16 genes used in qPCR validation. (XLSX 10 kb)

## Data Availability

All data sustaining the results in this study are included in this article or its supplemtary information files. Other datasets generated during this study are available upon resonable request from the corresponding author (Sabine Banniza).

## Abbreviations

DEG: Differentially expressed gene
DGE: Differential gene expression
FPKM: Fragments per kilobase of transcript per million
GO: Gene ontology
HPI: Hours post inoculation
HR: Hypersensitive response
PCA: Principal component analysis
PCD: Programmed cell death
PVCA: Principal variance component analysis
QTL: Quantitative trait loci
RIL: Recombination inbred line
RNA-Seq: RNA-sequencing
ROS: Reactive oxygen species
SB: Stemphylium blight
