# Supplementary figures and images for: Using a transcriptome sequencing approach to explore candidate resistance genes against stemphylium blight in the wild lentil species Lens ervoides
Source: BMC Plant Biol. 2019 Sep 11;19:399. doi: 10.1186/s12870-019-2013-6 (PMC6740027; doi:10.1186/s12870-019-2013-6)

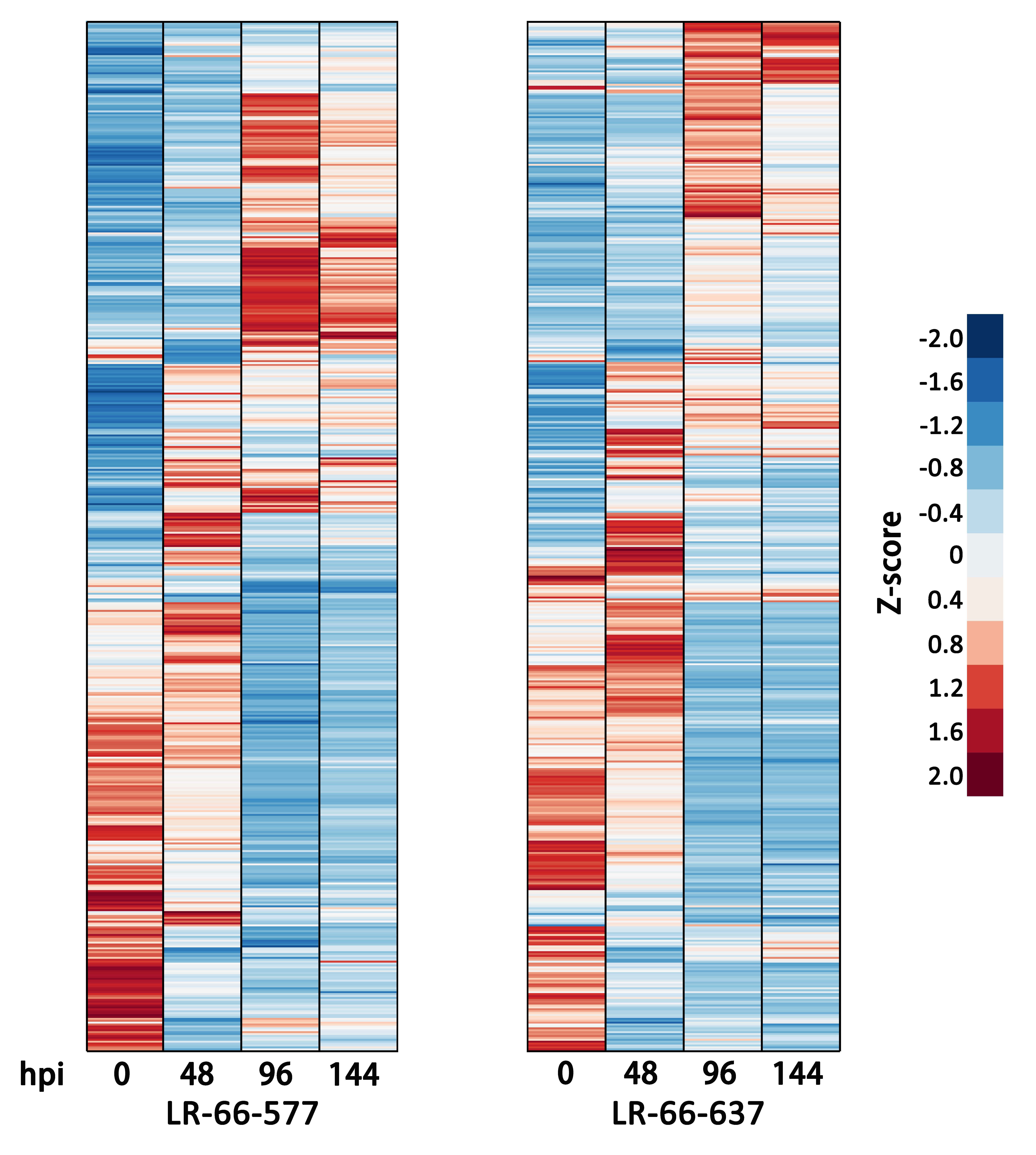

Supplement: Supplementary file 1 — Figure S1. Expression heatmap of 8810 disease-responsive genes for resistant Lens ervoides RIL LR-66-637 and susceptible RIL LR-66-577 at 0, 48, 96 and 144 hpi with Stemphylium botryosum (isolate SB19). (JPG 2425 kb) [file 12870_2019_2013_MOESM1_ESM.jpg]

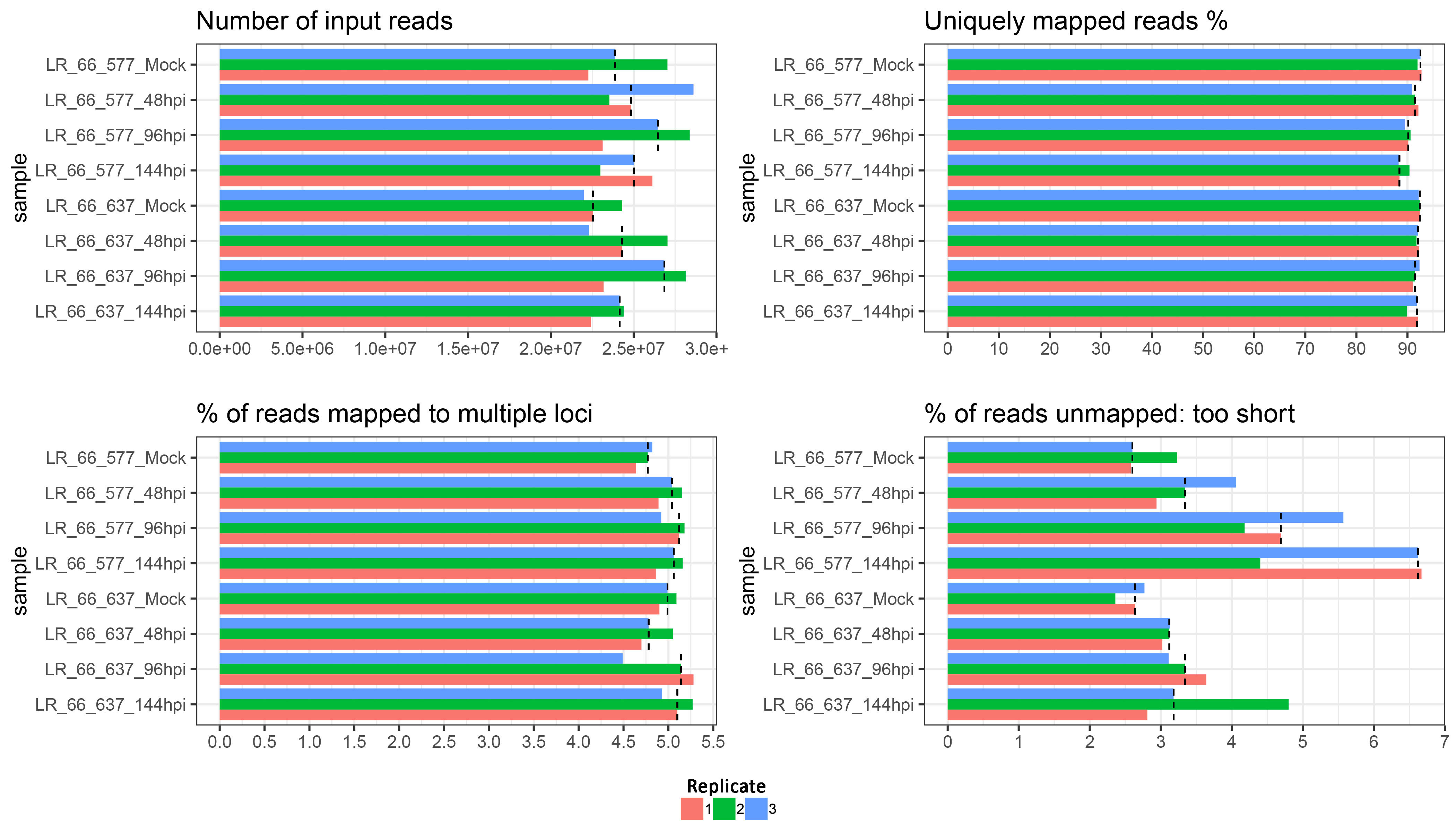

Supplement: Supplementary file 9 — Figure S2. Summary of mapping of 24 libraries on Lens culinaris reference genome. (JPG 1023 kb) [file 12870_2019_2013_MOESM9_ESM.jpg]
